# *In Vivo* Anti-Tumor Activity and Toxicological Evaluations of Perillaldehyde 8,9-Epoxide, a Derivative of Perillyl Alcohol

**DOI:** 10.3390/ijms17010032

**Published:** 2016-01-04

**Authors:** Luciana Nalone Andrade, Ricardo Guimarães Amaral, Grace Anne Azevedo Dória, Cecília Santos Fonseca, Tayane Kayane Mariano da Silva, Ricardo Luiz Cavalcante Albuquerque Júnior, Sara Maria Thomazzi, Lázaro Gomes do Nascimento, Adriana Andrade Carvalho, Damião Pergentino de Sousa

**Affiliations:** 1 Departamento de Fisiologia, Universidade Federal de Sergipe, CEP 49100-000, São Cristóvão, Sergipe CP 353, Brazil; lulisynalone@yahoo.com.br (L.N.A.); ricardoamaral23@hotmail.com (R.G.A.); gracedoria@hotmail.com (G.A.A.D.); cecilia_farma@hotmail.com (C.S.F.); tayane.kayane@yahoo.com.br (T.K.M.S.); sthomazzi@gmail.com (S.M.T.); 2 Laboratório de Morfologia e Patologia Experimental, Instituto de Tecnologia e Pesquisa, CEP 49032-490, Aracaju, Sergipe CP 203, Brazil; ricardo.patologia@uol.com.br; 3 Departamento de Ciências Farmacêuticas, Universidade Federal da Paraíba, CEP 58051-970, João Pessoa, Paraíba CP 5009, Brazil; lazarofarm2@gmail.com (L.G.N.); damiao_desousa@yahoo.com.br (D.P.S.); 4 Núcleo de Farmácia, Universidade Federal de Sergipe, CEP 49400-000, Lagarto, Sergipe CP 353, Brazil

**Keywords:** *p*-menthanes, monoterpenes, essential oils, natural products, antitumor activity, sarcoma 180, toxicity, anticancer, perillyl alcohol

## Abstract

Recent studies have revealed the high cytotoxicity of *p*-menthane derivatives against human tumor cells. In this study, the substance perillaldehyde 8,9-epoxide, a *p*-menthane class derivative obtained from (*S*)-(−)-perillyl alcohol, was selected in order to assess antitumor activity against experimental sarcoma 180 tumors. Toxicological effects related to the liver, spleen, kidneys and hematology were evaluated in mice submitted to treatment. The tumor growth inhibition rate was 38.4%, 58.7%, 35.3%, 45.4% and 68.1% at doses of 100 and 200 mg/kg/day for perillaldehyde 8,9-epoxide, perillyl alcohol and 25 mg/kg/day for 5-FU intraperitoneal treatments, respectively. No toxicologically significant effect was found in liver and kidney parameters analyzed in Sarcoma 180-inoculated mice treated with perillaldehyde 8,9-epoxide. Histopathological analyses of the liver, spleen, and kidneys were free from any morphological changes in the organs of the animals treated with perillaldehyde 8,9-epoxide. In conclusion, the data suggest that perillaldehyde 8,9-epoxide possesses significant antitumor activity without systemic toxicity for the tested parameters. By comparison, there was no statistical difference for the antitumor activity between perillaldehyde 8,9-epoxide and perillyl alcohol.

## 1. Introduction

Cancer is a complex genetic disease comprising specific characteristics that causes mortality in both children and adults, triggering a global health concern. More than 100 distinct types and subtypes of cancer can be found within specific organs [1,2,3]. It is a multifactorial condition causing both uncontrolled growth and invasion of abnormal cells, and which eventually leads to tumor formation [4]. The major problems in treating cancer are the loss of sensitivity of tumor cells to the several chemotherapeutic agents and their severe adverse effects, leading to the search for alternative treatments [5].

The research on derivatives of natural products has revealed new possibilities for therapeutic anticancer agents. More than two thirds of the drugs currently used in cancer treatments come directly from natural products, or have been developed using knowledge gained from the activities of their ingredients [6,7,8]. Attempting to obtain more potent drugs, many studies with natural products and their analogues have been conducted, showing antitumor properties of various plants and their constituents [9,10].

Monoterpenoid compounds with *p*-menthane structures are abundantly found in nature. [11]. Several of these compounds, such as carvacrol [12], thymol [13,14], d-limonene [15], perillic acid [16,17], perillaldehyde [18], and perillyl alcohol [19,20], have been studied for their anticancer potential. Others have also been reported to possess *in vitro* cytotoxic effects on cancer cell lines [9]. The monoterpene perillyl alcohol is the most promising member of the group *p*-menthanes. Perillyl alcohol was found to be cytotoxic to a wide variety of cancer cells through advanced studies demonstrating its bioactivity as an antitumor agent [21,22,23,24,25,26,27].

The compound perillaldehyde 8,9-epoxide is a synthetic derivative of structurally correlated perillyl alcohol [28]. In a study of the percentage growth inhibition of cells (GI%) by Andrade and collaborators [28], perillyl alcohol and its derivative perillaldehyde 8,9-epoxide were evaluated against tumor cell lines of ovarian adenocarcinoma, colon carcinoma, and glioblastoma; a higher *in vitro* GI% for perillaldehyde 8,9-epoxide was demonstrated. The study indicated that the presence of epoxide and aldehyde functions may be a determinant for high cytotoxicity.

**Figure 1:**
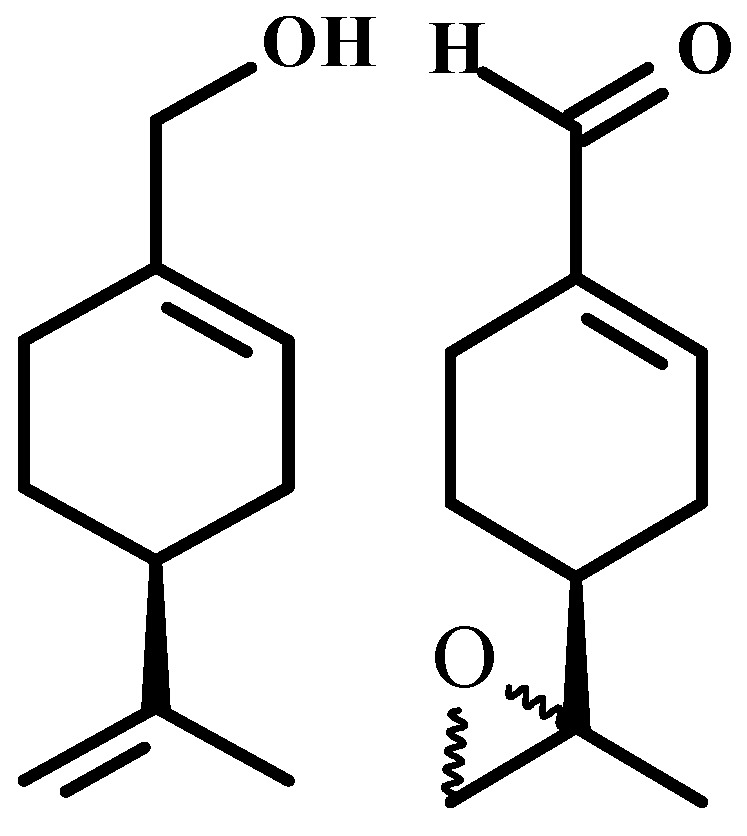
Chemical structures of (*S*)-(−)-perillyl alcohol and perillaldehyde 8,9-epoxide.

In light of the promising results found in clinical trials with perillyl alcohol’s derivative high cytotoxic activity perillaldehyde 8,9-epoxide, and with the understanding that structural change can reveal new possibilities for the treatment of cancer, this study aimed to assess the antitumor activity and toxicological effects of the derivative of perillyl alcohol perillaldehyde 8,9-epoxide (Figure 1) in an experimental model using Sarcoma 180-inoculated mice.

## 2. Results and Discussion

### 2.1. In Vivo Antitumor Evaluation of Perillaldehyde 8,9-Epoxide

The compound perillaldehyde 8,9-epoxide is a derivative of perillyl alcohol, and belongs to the *p*-menthane class. In a recent study, the cytotoxic potential of eighteen *p*-menthane derivatives was investigated, and it was demonstrated that the perillaldehyde 8,9-epoxide exhibits high cytotoxic activity for 4 cancer cell lines (colon carcinoma, ovarian adenocarcinoma, glioblastoma, and leukemia), with an median inhibitory concentration able to induce 50% of maximal effect (IC_50_) of less than 4.0 µg/mL for all of the cell lines studied [28]. According to the National Cancer Institute (Bethesda, MD, USA) in its selection program of anticancer drugs, IC_50_ values less than or equal to 4.0 µg/mL are considered anticancer agent candidates [29]. So, due to the promising activity, we decided to investigate the effects of perillaldehyde 8,9-epoxide *in vivo*.

**Figure 2:**
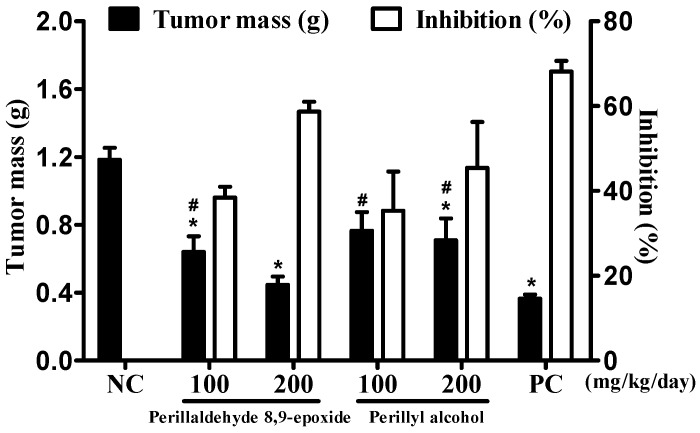
The antitumor activity of perillaldehyde 8,9-epoxide and perillyl alcohol on animals inoculated with sarcoma 180 tumor cells (S-180). The graphs show tumor mass (g) and tumor growth inhibition rate (%). Administration of the compounds was performed intraperitoneally, at doses of 100 and 200 mg/kg/day, starting 24 h after the inoculation of S-180, during 7 consecutive days. As a positive control (PC), we used 5-Fluorouracil (25 mg/kg/day, i.p.). As a negative control (NC), we used 5% DMSO. Data are presented as mean ± SEM (*n* = 10 animals/group). * *p* < 0.05 compared with the NC group, and # *p* < 0.05 compared with the PC group by variance analysis, followed by the Student-Newman-Keuls test.

The effects of perillaldehyde 8,9-epoxide and perillyl alcohol on the sarcoma 180 tumor cells (S-180) into mice are presented in Figure 2. Twenty four hours after the last treatment day, the mean tumor mass of the negative control (NC) in animals was found to be 1.18 ± 0.07 g. In the presence of the intraperitoneal administrated compounds (100 and 200 mg/kg/day), the mean tumor masses were 0.64 ± 0.03 and 0.45 ± 0.02 g for perillaldehyde 8,9-epoxide, and 0.76 ± 0.10 and 0.70 ± 0.12 g for perillyl alcohol, respectively. At both doses the perillaldehyde 8,9-epoxide and perillyl alcohol groups were statistically different from the NC group (*p* < 0.05). Tumor growth inhibition rates were 38.4% and 58.7% for the perillaldehyde 8,9-epoxide and 35.3% and 45.4% for the perillyl alcohol at doses of 100 and 200 mg/kg/day, respectively. Therefore, both 8,9-epoxide perillaldehyde and perillyl alcohol exhibited antitumor activity in two doses, with a higher inhibition rate at a dose of 200 mg/kg/day.

Positive control (PC) reduced tumor mass by 0.36 ± 0.02 g with a tumor growth inhibition rate of 68.1%. When comparing the PC group to the groups treated with perillaldehyde 8,9-epoxide and perillyl alcohol at both doses, statistical changes were observed for all the groups (*p* < 0.05). Both the tested compounds show antitumor activity, but not as intense as those of 5-FU (25 mg/kg/day).

Several studies have shown that many *p*-menthane monoterpenes have *in vitro* and *in vivo* antitumor activity*.* Examples are linalyl acetate, α-terpineol, carvone, menthol, terpinene, thymol, thymoquinone, sobrerol, limonene, perillic acid, perillyl alcohol, and perillaldehyde. These results are important and provide insights as to their use for cancer treatments [23].

Among monoterpenes, perillyl alcohol stands out for its cytotoxic activity, and clinical trials in phase I and II have been performed to develop this compound as a pharmaceutical for intranasal administration treatment of gliomas [16,30]. However, the comparative study of perillyl alcohol and perillaldehyde 8,9-epoxide against three human tumor cell lines *in vitro* showed that perillaldehyde 8,9-epoxide exhibited a higher inhibition percentage in all cell lines tested as compared to perillyl alcohol [28]. In the present study, comparing perillaldehyde 8,9-epoxide with perillyl alcohol demonstrated that there was no significant difference between the compounds (*p* > 0.05). Therefore, both compounds showed similar pharmacological efficacy against S-180 tumor *in vivo*. Thus, similarly to perillyl alcohol, perillaldehyde 8,9-epoxide becomes a compound of interest in research of new drugs against cancer.

### 2.2. Systemic Toxicological Evaluation

Systemic toxicological evaluation of antitumor drug activity is very important because most chemotherapeutic cancer agents are non-specific in action; besides attacking tumors, they rapidly demolish normally dividing cells, and can cause extensive collateral side effects [31]. Therefore, the evaluation of systemic toxicological effects was performed on animals submitted to treatment with perillaldehyde 8,9-epoxide, as shown in the Table 1, Table 2 and Table 3.

**Table 1:** The effect of perillaldehyde 8,9-epoxide on the body and organ weights of mice transplanted with S-180 tumor cells.

Treatment	Dose (mg/kg/Day)	Variation Body in Mass (g)	Liver (g/100 g Body Mass)	Spleen (g/100 g Body Mass)	Kidney (g/100 g Body Mass)
**Heathy mice**					
Negative control (5% DMSO)	–	0.14 ± 0.08	4.76 ± 0.13	0.36 ± 0.01	1.32 ± 0.05
Positive control	25	−3.50 ± 0.66 *	4.65 ± 0.21	0.19 ± 0.02 *	1.13 ± 0.03
Perillaldehyde	100	0.90 ± 0.38	4.66 ± 0.09	0.25 ± 0.01	1.15 ± 0.06
8,9-epoxide	200	−0.71 ± 0.53	5.07 ± 0.13	0.37 ± 0.06	1.17 ± 0.19
**Mice with tumor S-180**					
Negative control (5% DMSO)	–	0.61 ± 0.20	4.63 ± 0.09	0.49 ± 0.03	1.14 ± 0.02
Positive control	25	−4.46 ± 0.83 ^#^	4.99 ± 0.16	0.33 ± 0.02 ^#^	1.15 ± 0.04
Perillaldehyde	100	1.11 ± 0.19	4.84 ± 0.07	0.52 ± 0.02	1.27 ± 0.03
8,9-epoxide	200	−1.58 ± 0.33	4.57 ± 0.09	0.49 ± 0.04	1.28 ± 0.03

Data are presented as mean ± SEM (*n* = 10 animals/group with tumor S-180, and *n* = 5 animals/group heathy mice). * *p* < 0.05 for all groups of healthy mice compared with the negative control group of heathy mice and ^#^ *p* < 0.05 for all groups of mice with tumor S-180 compared with the control tumor S-180 group using analysis of variance, followed by the Student-Newman-Keuls test.

**Table 2:** The effect of perillaldehyde 8,9-epoxide on the biochemical parameters determined through blood of healthy animals and those inoculated with S-180 tumor cells.

Drug	Dose (mg/kg/Day)	AST (U/L)	ALT (U/L)	Urea (mg/dL)	Creatinine (mg/dL)
**Healthy mice**					
Negative control (5% DMSO)	–	82.5 ± 3.48	52.50 ± 4.80	64.75 ± 4.31	0.39 ± 0.01
Positive control	25	102.0 ± 15.06	47.25 ± 2.8	49.00 ± 5.86	0.37 ± 0.02
Perillaldehyde	100	100.5 ± 5.97	55.25 ± 6.26	54.25 ± 3.82	0.34 ± 0.01
8,9-epoxide	200	78.75 ± 3.17	47.75 ± 4.64	41.00 ± 4.42	0.34 ± 0.01
**Mice with S-180 tumor cells**					
Negative control (5% DMSO)	–	181.2 ± 12.6	34.6 ± 1.5	55.20 ± 1.81	0.34 ± 0.02
Positive control	25	164.4 ± 10.0	42.8 ± 0.6	50.71 ± 2.18	0.28 ± 0.09
Perillaldehyde	100	178.0 ± 5.0	35.0 ± 2.2	43.0 ± 1.08	0.34 ± 0.01
8,9-epoxide	200	208.3 ± 20.6	36.0 ± 3.5	47.80 ± 2.01	0.33 ± 0.02

AST: aspartate aminotransferase; ALT: alanine aminotransferase. Data are presented as mean ± SEM *n* = 5 animals/group.

To evaluate body mass variations, the animals were weighed at the start and finish of the experiment (Table 1). No significant changes were observed in corporal mass of mice after administration of perillaldehyde 8,9-epoxide in either the healthy mice or the S-180 tumor group. The mass of each organ was expressed as grams per 100 grams of corporal mass, and the healthy control group was compared with the S-180 tumor group (Table 1). No significant changes in the masses of livers, spleens and kidneys in groups of animals treated with perillaldehyde 8,9-epoxide were observed. The positive control group (5-Fluorouracil, 25 mg/kg/day) demonstrated a decrease in body mass and in the mass of the spleen (*p* < 0.05) in both the healthy mice and in the S-180 tumor group, which corroborates findings in the literature [32]. The fact that perillaldehyde 8,9-epoxide did not cause changes in body or organ masses is a positive result, since changes are often found in compounds with antitumor activity [8]. The loss of body mass is a major effect of anti-neoplastics as a result of the severe nausea and vomiting often caused by the medication used, which may cause intense discomfort to the patient [33].

**Table 3:** The effect of perillaldehyde 8,9-epoxide on the hematological parameters determined through blood of healthy animals and those with S-180 tumor cells.

Treatment	Dose (mg/kg/Day)	Total Leukocytes (10^3^ Cells/µL)	Differential Count of Leukocytes (%)			
			Eosinophil	Lymphocyte	Neutrophil	Monocyte
**Healthy mice**						
Negative control (5% DMSO)	-	7.78 ± 0.98	0	72.5 ± 2.08	25.8 ± 5.43	1.7 ± 0.64
Positive control	25	2.37 ± 0.77 *	0	95.8 ± 0.70 *	3.0 ± 0.70 *	1.2 ± 0.70
Perillaldehyde	100	3.12 ± 0.38 *	0	86.0 ± 1.68 *	12.5 ± 1.08 *	1.5 ± 0.86
8,9-epoxide	200	2.62 ± 0.47 *	0	89.5 ± 0.65 *	9.2 ± 0.85 *	1.3 ± 0.25
**Mice with S-180 tumor**						
Negative control (5% DMSO)	-	11.20 ± 1.15	0	54.2 ± 2.61	44.6 ± 1.69	1.2 ± 0.58
Positive control	25	2.50 ± 0.32 ^#^	0	89.0 ± 0.70 ^#^	10.2 ± 0.54 ^#^	0.8 ± 0.37
Perillaldehyde	100	5.30 ± 0.37 ^#^	0	73.2 ± 2.17 ^#^	26.2 ± 5.92 ^#^	0.6 ± 0.40
8,9-epoxide	200	6.50 ± 0.65 ^#^	0	80.6 ± 2.54 ^#^	18.6 ± 6.18 ^#^	0.8 ± 0.34

Data are presented as mean ± SEM (*n* = 05 animals/group). * *p* < 0.05 for all groups of healthy mice compared with the negative control group of healthy mice and ^#^ *p* < 0.05 for all groups of mice with tumor S-180 compared with the negative control group tumor S-180 using analysis of variance, followed by the Student-Newman-Keuls test.

#### 2.2.1. The Effect of Perillaldehyde 8,9-Epoxide on Biochemical Parameters

The liver and kidneys are the most important organs for detoxification and excretion, respectively. Most anticancer drugs generate various toxic effects such as nephrotoxicity and hepatotoxicity. Irinotecan and mithramycin causing liver problems and paclitaxel induced renal damage are examples of these toxic effects [34]. With this in mind, we performed biochemical tests to assess liver function (ALT and AST), and renal activity (urea and creatinine).

Table 2 shows no statistically significant changes in the parameters tested comparing the control (negative control) group of healthy mice with healthy treated groups (*p* > 0.05). When comparing treated mice (inoculated with S-180 tumor) to the control S-180 mice group there was also no statistically significant changes. These results indicate that perillaldehyde 8,9-epoxide does not cause changes in hepatic and renal parameters at the doses tested within the treatment time frame.

Interestingly, the AST values presented by the groups of mice inoculated with S-180 are higher than the values of the healthy mice groups. This result is justified by the fact that AST is also found in skeletal and cardiac muscles, kidneys, pancreas, and erythrocytes [35]. S-180 tumor, in solid form, grows in axillary regions, possibly causing tissue damages in the skeletal muscle and liver, and consequently leading to changes in the AST and ALT enzymatic biochemical parameter results.

#### 2.2.2. The Effect of Perillaldehyde 8,9-Epoxide on Hematological Parameters

Most anti-neoplastics generate various toxic effects and immunosuppression, which result from the lack of drug specificity [34]. Hematological parameters, including the total count as well as differential counts of leukocytes, including eosinophils, lymphocytes, neutrophils and monocytes, were evaluated in the blood of healthy mice and those with tumor S-180, as shown in Table 3.

In blood (after intraperitoneal administration) from the animals inoculated with S-80 tumor cells, both perillaldehyde 8,9-epoxide and PC induced decreases in the total leukocyte counts when compared to the negative control group (with S-180 tumor cells) (*p* < 0.05). Also in the peripheral blood of healthy mice, perillaldehyde 8,9-epoxide and 5-FU induced decreases in total leukocytes when compared to the healthy negative control group (*p* < 0.05). These results suggest that perillaldehyde 8,9-epoxide caused leukopenia at the doses tested and at the applied time of treatment. One of the serious problems of the currently available anticancer drugs on the market is the decrease in the number of total leukocytes, which increases the susceptibility of patients to infections. An example of this feature is the 5-FU used as positive control in this study [36,37]. Unfortunately perillaldehyde 8,9-epoxide does not escape this feature.

In the blood cell differential count, we also observed significant changes in the percentage of neutrophils and lymphocytes when comparing healthy treated groups with the healthy negative control group. The same change was observed when comparing S-180 treated groups with the S-180 (negative control alone) injected group (Table 3). This demonstrates that in addition to leukopenia, perillaldehyde 8,9-epoxide caused changes in the percentages of lymphocytes and neutrophils.

#### 2.2.3. Histopathological Analyses

**Figure 3:**
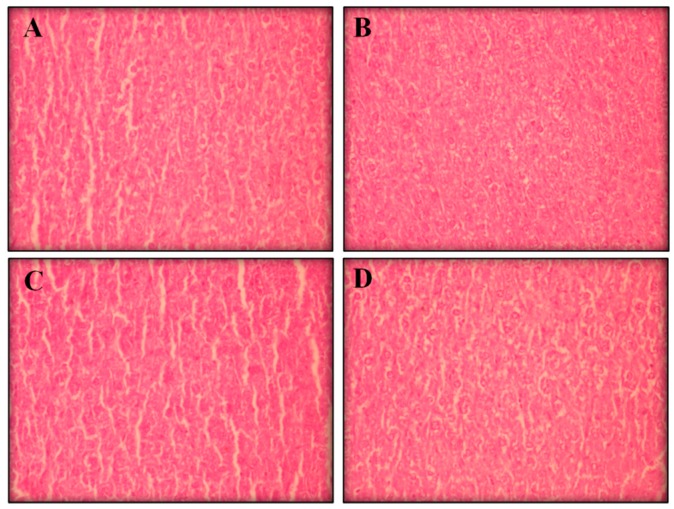
The effect of perillaldehyde 8,9-epoxide on livers of healthy mice. Histopathology was analyzed by light microscopy and stained with hematoxylin-eosin (400×). Photos from liver represent negative control (**A**); positive control 25 mg/kg/day 5-FU-treated (**B**); 100 mg/kg/day perillaldehyde 8,9-epoxide treated (**C**); and 200 mg/kg perillaldehyde 8,9-epoxide treated (**D**).

**Figure 4:**
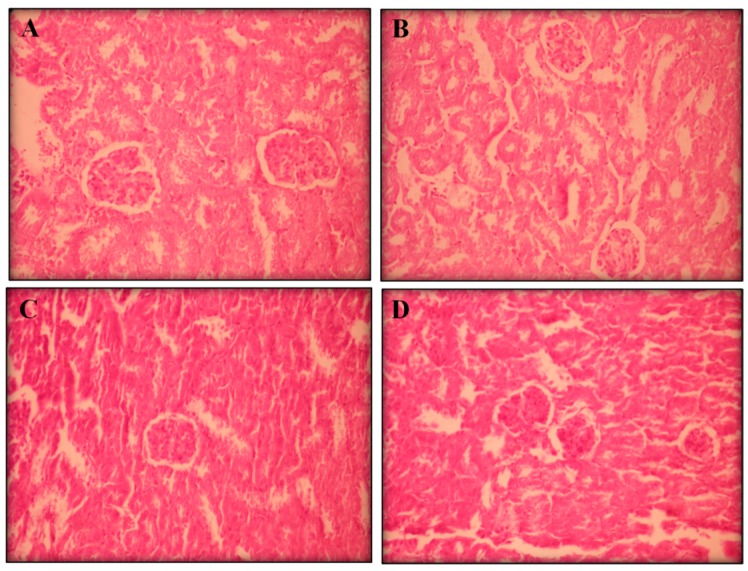
The effect of perillaldehyde 8,9-epoxide on kidneys of healthy mice. Histopathology was analyzed by light microscopy and stained with hematoxylin-eosin (400×). Photos from liver represent: negative control (**A**); positive control 25 mg/kg/day 5-FU-treated (**B**); 100 mg/kg/day perillaldehyde 8,9-epoxide treated (**C**); and 200 mg/kg perillaldehyde 8,9-epoxide treated (**D**).

**Figure 5:**
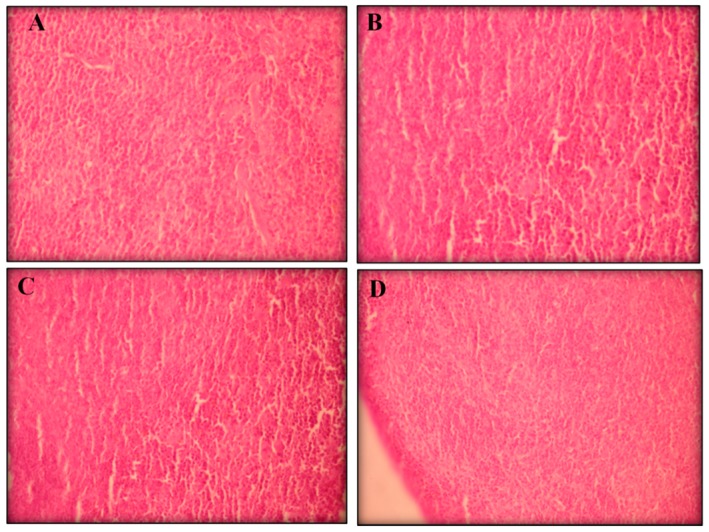
The effect of perillaldehyde 8,9-epoxide on spleens of healthy mice. Histopathology was analyzed by light microscopy and stained with hematoxylin-eosin (400×). Photos from liver represent: negative control (**A**); positive control 25 mg/kg/day 5-FU-treated (**B**); 100 mg/kg/day perillaldehyde 8,9-epoxide treated (**C**); and 200 mg/kg perillaldehyde 8,9-epoxide treated (**D**).

Visible atrophy was observed in the spleens of the mice treated with positive control. Histopathological analyses of the livers, kidneys, and spleens (removed from all groups) showed no remarkable changes in the morphology of their tissues (Figure 3, Figure 4 and Figure 5, respectively).

**Figure 6:**
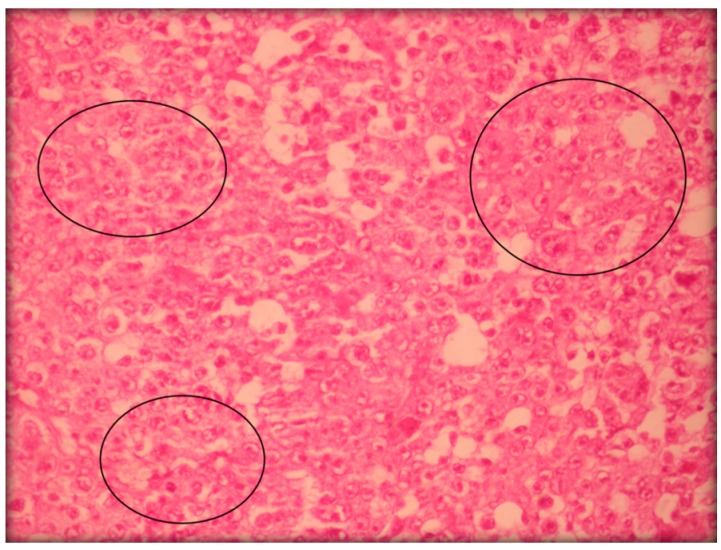
Histopathology of Sarcoma 180 tumor cells was analyzed by light microscopy and stained with hematoxylin-eosin (400×). Circles show the presence of mitosis.

Figure 6 shows histopathology of Sarcoma 180 tumor cells.

## 3. Experimental Section

### 3.1. Drug

In a 250 mL flask containing a solution of perillaldehyde (1.0 g, 6.66 mmol) in CH_2_Cl_2_ (40 mL), a solution of meta-chloroperoxybenzoic acid (*m*-CPBA) (70% 1.81 g, 7.37 mmol) in dry CH_2_Cl_2_ (40 mL) was added slowly and kept at 0 °C (ice bath), as described by Kido and collaborators [38]. The reaction medium was maintained under stirring for a 4 h period at the same temperature. Afterwards it was removed from the ice bath and added to the reaction mixture with aqueous 10% NaHSO_3_ (50 mL). The aqueous phase was extracted with CH_2_Cl_2_ (50 mL), and the combined organic phases were washed with a 5% NaHCO_3_ solution (50 mL), and dried with anhydrous Na_2_SO_4_. The solvent was then concentrated on a rotary evaporator. The product was purified by column chromatography on silica gel (hexane/ethyl acetate 9:1). Perillaldehyde 8,9-epoxide was obtained with a 48.5% (3.20 mmol) yield. 

Perillaldehyde 8,9-epoxide is a *p*-menthane compound that has a molecular formula of C_10_H_14_O_2_, molar volume of 140.6 ± 3.0 cm^3^/mol, surface tension of 29.6 ± 3.0 dyn/cm, and density of 1.181 ± 0.06 g/cm. The compound perillaldehyde 8,9-epoxide was analyzed by infrared, ^1^H and ^13^C NMR techniques.

### 3.2. Animals

Eighty Swiss mice (females, weight varying between 25 and 30 g) were obtained from the central bioterium of the Federal University of Sergipe, Brazil. The animals were housed and kept under a temperature-controlled room (22–25 °C), with 12:12 h light-dark cycle and free access to food and water. The experimental protocol was submitted and approved by the Animal Care and Use Committee at the Federal University of Sergipe (CEPA: 16/2014).

### 3.3. In Vivo Antitumor Activity Assay

#### 3.3.1. Determination of the Effect of Perillaldehyde 8,9-Epoxide on *in Vivo* Tumor Growth

Sarcoma 180 (S-180) tumor cells, were obtained through at the Federal University of Ceará, Brazil and maintained in the abdominal cavities of Swiss mice in their ascitic form by ten days.

The effect on tumor growth *in vivo* was assessed using S-180 ascites tumor cells, according standard protocol [34,39]. Ten-day-old S-180 ascites tumor cells (2 × 10^6^ cells per 0.5 mL) were inoculated subcutaneously into the right axillary region of animals. After 24 h, the animals were separated into 5 groups (*n* = 10 mice/group) and subjected to intraperitoneal (i.p.) treatment for 7 consecutive days (once a day). Group 1 consisted of animals treated by intraperitoneal (i.p.) administration of negative control—5% Dimethyl sulfoxide (DMSO); Group 2 consisted of animals treated by i.p. administration of positive control—5-fluorouracil 25 mg/kg/day (5-FU; purity > 99%; Sigma Chemical Co., St. Louis, MO, USA); Group 3 consisted of animals treated by i.p. administration of perillaldehyde 8,9-epoxide at 100 mg/kg/day; Group 4 consisted of animals treated by i.p. administration of perillaldehyde 8,9-epoxide at 200 mg/kg/day; Group 5 consisted of animals treated by i.p. administration of perillyl alcohol at 100 mg/kg/day; Group 6 consisted of animals treated by i.p. administration of perillyl alcohol at 200 mg/kg/day. At 24 h from the last day of treatment, under 1.5% isoflurane inhalation anesthesia, the mice were euthanized, and the tumors were weighed. The inhibition ratio of tumor growth (%) was calculated by the following equation: (%) = [(*NC* − *TG*)/*NC*] × 100, where *NC* is the average tumor weight of the negative control group and *TG* is the average tumor weight of the treated group [39].

#### 3.3.2. Systemic Toxicological Assessment

##### Determination of the Effect of Perillaldehyde 8,9-Epoxide on Body and Organ Weight

Body weights (*n* = 10 mice/group) were determined at the first and last days of treatment. Organ weight alterations (*n* = 10 mice/group), and hematological and biochemical analysis (*n* = 5 mice/group) were performed on the last day of experimentation. All systemic toxicological analyses were also performed with 4 healthy animal groups, without the inoculation of S-180, (*n* = 5 mice/group) treated with 5% DMSO (i.p.), 5-FU 25 mg/Kg/day (i.p.), perillaldehyde 8,9-epoxide 100 mg/kg/day (i.p.) and perillaldehyde 8,9-epoxide 200 mg/kg/day for consecutive 7 days.

Twenty four hours after the seventh treatment day, blood samples of the animals were collected (retro-orbital plexus) with anesthetized mice (1.5%, isoflurane). Immediately after blood collection the animals were euthanized, and their organs (liver, spleen and kidneys) were removed and weighed. The wet mass of each organ was expressed as grams per 100 grams of body mass and compared to the negative control group.

##### Determination of the Effect of Perillaldehyde 8,9-Epoxide on Biochemical Parameters

For evaluation of the effect of perillaldehyde 8,9-epoxide on the biochemical parameters aspartate aminotransferase (AST) and alanine aminotransferase (ALT) enzymatic to investigate liver function alterations, creatinine and urea were measured as renal function parameters. For this test, blood samples of the animals were collected without anticoagulant, centrifuged for 10 min at 3500 rpm and 25 °C to obtain plasma.

The Clinical Chemistry^®^ kits (Abbott Architect; C8000; Abbott Laboratories, Wiesbaden, Germany) were used for biochemical evaluation of serum samples.

##### Determination of the Effect of Perillaldehyde 8,9-Epoxide on Hematological Parameters

For hematological analyses, an aliquot of blood from each animal was placed in ethylene diamine tetracetic acid (EDTA). Hematological parameters, including the total as well as differential counts of leukocytes, including eosinophils, lymphocytes, neutrophils and monocytes determined by standard manual procedures using optical light microscopy.

##### Histopathological Analyses

After being weighed and fixed in 10% formaldehyde; tumors, livers, spleens, and kidneys were submitted for assessment macroscopic (size, coloration and hemorrhage), were cut into small pieces and, after that dehydrated in alcohol, diaphanized in xylene and paraffin-embedded. Subsequently, 5-µm-thick histological sections were obtained and stained with hematoxylin and eosin. Histological analyses were performed by light microscopy in order to assess possible damage caused by treatment.

##### Statistical Analysis

Data obtained were expressed as the mean ± SEM and the differences among experimental groups were evaluated using one-way analysis of variance ANOVA followed by the Student Newman-Keuls test. Were considered significant values of *p* < 0.05. All statistical analyses were carried using the GraphPad program (Intuitive Software for Science, San Diego, CA, USA).

## 4. Conclusions

In conclusion, the data presented demonstrate the *in vivo* antitumor activity of perillaldehyde 8,9-epoxide without alterations in biochemical or histopathological parameters. Additionally, perillaldehyde 8,9-epoxide showed similar antitumor potency to perillyl alcohol. Further studies using perillaldehyde 8,9-epoxide and other structurally similar compounds may well be carried out against other tumor types.
